# Gene-Silencing Therapeutic Approaches Targeting PI3K/Akt/mTOR Signaling in Degenerative Intervertebral Disk Cells: An In Vitro Comparative Study Between RNA Interference and CRISPR–Cas9

**DOI:** 10.3390/cells13232030

**Published:** 2024-12-09

**Authors:** Masao Ryu, Takashi Yurube, Yoshiki Takeoka, Yutaro Kanda, Takeru Tsujimoto, Kunihiko Miyazaki, Hiroki Ohnishi, Tomoya Matsuo, Naotoshi Kumagai, Kohei Kuroshima, Yoshiaki Hiranaka, Ryosuke Kuroda, Kenichiro Kakutani

**Affiliations:** Department of Orthopaedic Surgery, Kobe University Graduate School of Medicine, 7-5-1 Kusunoki-cho, Chuo-ku, Kobe 650-0017, Japan; masaoryu0724@gmail.com (M.R.); yoshiki_tkk@hotmail.com (Y.T.); ykanda221@gmail.com (Y.K.); t.1105.tsujimoto@gmail.com (T.T.); miya625819@gmail.com (K.M.); o0717ooo@yahoo.co.jp (H.O.); t.matsuo512@gmail.com (T.M.); kumagaiisumi19891107@gmail.com (N.K.); koheikuroshima@gmail.com (K.K.); yoshiagain@gmail.com (Y.H.); kurodar@med.kobe-u.ac.jp (R.K.); kakutani@med.kobe-u.ac.jp (K.K.)

**Keywords:** intervertebral disk, disk degeneration, nucleus pulposus (NP) cells, phosphatidylinositol 3-kinase (PI3K)/Akt/mammalian target of rapamycin (mTOR) signaling, autophagy, RNA interference (RNAi), small interfering RNA (siRNA), clustered regularly interspaced short palindromic repeat (CRISPR)–CRISPR-associated protein 9 (Cas9), gene-silencing therapy, spine

## Abstract

The mammalian target of rapamycin (mTOR), a serine/threonine kinase, promotes cell growth and inhibits autophagy. The following two complexes contain mTOR: mTORC1 with the regulatory associated protein of mTOR (RAPTOR) and mTORC2 with the rapamycin-insensitive companion of mTOR (RICTOR). The phosphatidylinositol 3-kinase (PI3K)/Akt/mTOR signaling pathway is important in the intervertebral disk, which is the largest avascular, hypoxic, low-nutrient organ in the body. To examine gene-silencing therapeutic approaches targeting PI3K/Akt/mTOR signaling in degenerative disk cells, an in vitro comparative study was designed between small interfering RNA (siRNA)-mediated RNA interference (RNAi) and clustered regularly interspaced short palindromic repeat (CRISPR)–CRISPR-associated protein 9 (Cas9) gene editing. Surgically obtained human disk nucleus pulposus cells were transfected with a siRNA or CRISPR–Cas9 plasmid targeting *mTOR*, *RAPTOR*, or *RICTOR*. Both of the approaches specifically suppressed target protein expression; however, the 24-h transfection efficiency differed by 53.8–60.3% for RNAi and 88.1–89.3% for CRISPR–Cas9 (*p* < 0.0001). Targeting *mTOR*, *RAPTOR*, and *RICTOR* all induced autophagy and inhibited apoptosis, senescence, pyroptosis, and matrix catabolism, with the most prominent effects observed with *RAPTOR* CRISPR–Cas9. In the time-course analysis, the 168-h suppression ratio of RAPTOR protein expression was 83.2% by CRISPR–Cas9 but only 8.8% by RNAi. While RNAi facilitates transient gene knockdown, CRISPR–Cas9 provides extensive gene knockout. Our findings suggest that RAPTOR/mTORC1 is a potential therapeutic target for degenerative disk disease.

## 1. Introduction

Up to 85% of people experience low back pain at some point in their lifetime [1]. Low back pain leads to disability that can increase medical expenses and affect the workforce [1]. Healthcare costs associated with low back pain are approximately USD 100 billion annually in the USA [2]. The mechanism behind low back pain is multifactorial; however, intervertebral disk degeneration is recognized as one of the independent causes [3].

The intervertebral disk is structurally composed of the central nucleus pulposus (NP), surrounded by the peripheral annulus fibrosus and bordered by cartilage endplates. The disk is anatomically the largest avascular organ in the human body [4], to which the oxygen and nutrient supply depends on diffusion through the endplates. Endplate calcification and subchondral bone sclerosis occur with aging, which can limit this delivery. Such oxygen and nutrient deprivation is a suspected contributor to disk degeneration [5].

Intervertebral disk degeneration is biochemically characterized by the degradation of the extracellular matrix [6]. The regulation of the matrix metabolism depends on a dynamic equilibrium between enzymes promoting degradation and inhibitors counteracting these processes. Catabolic enzymes include matrix metalloproteinases (MMPs) and disintegrin and metalloproteinase with thrombospondin motifs (ADAMTSs), while anti-catabolic inhibitors include tissue inhibitors of metalloproteinases (TIMPs) [7]. Activated and increased MMP and ADAMTS levels relative to TIMPs are often observed in human clinical cases [8,9,10] and rodent experimental disk degeneration models [11,12,13]. This results in the degradation of matrix components, including proteoglycans, mainly aggrecan, and collagens, predominantly type II in the NP [6]. A reduction in the cell number is another key feature of intervertebral disk degeneration [14], which is attributed to programmed cell death, primarily apoptosis [15]. High apoptosis rates [16] have been observed in both human [17] and rodent disk aging and degeneration [13,18]. In addition, pyroptosis is an inflammatory form of cell death that releases cytokines, such as interleukin (IL)-1β and IL-18, driven by caspase-1 activation [19]. This cleaves gasdermin D (GSDMD) to form membrane pores and induce cell lysis [20,21]. In the intervertebral disk, particularly NP cells, pyroptosis promotes inflammation, extracellular matrix degradation, and cell death, which accelerates the degenerative process [22]. The incidence of irreversible cell growth arrest due to aging, known as senescence [23], is also elevated in human disk degeneration [24,25,26]. In addition, the involvement of autophagy, an intracellular self-degradation and recycling process [27], has garnered growing interest in the context of human [28,29] and animal [30,31] intervertebral disks. Disk cell apoptosis and senescence, as well as radiographic and histological degeneration, have been induced by the knockdown of key autophagy-related genes [29,31]. Evidence of autophagy has also declined with the severity of experimental disk degeneration in rats [32]. In fact, autophagy plays a critical role in cell survival by supporting metabolic homeostasis and removing misfolded proteins and damaged organelles, particularly under stress conditions such as hypoxia and nutrient scarcity [27]. At the molecular level, autophagy is tightly inhibited by the mammalian target of rapamycin (mTOR) through the phosphatidylinositol 3-kinase (PI3K)/Akt/mTOR axis [33]. Therefore, we hypothesized that resident disk cells can use autophagy and PI3K/Akt/mTOR signaling for adapting to the harsh, hypoxic, and nutrition-deprived environment.

**Figure 1:**
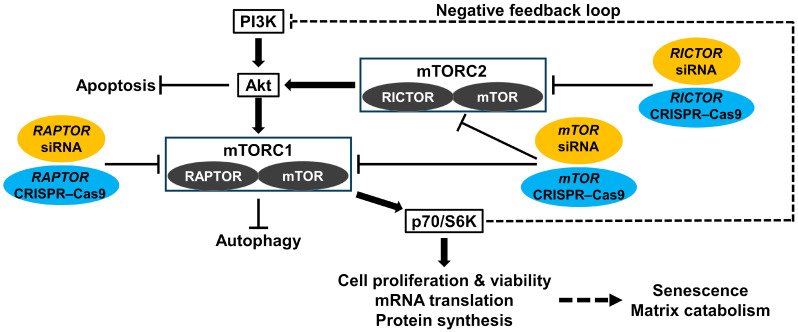
Schematic illustration of human disk intracellular PI3K/Akt/mTOR signaling pathway. The mTOR is a serine/threonine kinase that integrates nutrient signals to promote drive cell growth and division. It operates within the following two primary complexes: mTORC1 and mTORC2, which include RAPTOR and RICTOR, respectively. The downstream effectors of mTORC1, such as p70/S6K, are involved in controlling cell proliferation, mRNA translation, and protein synthesis, also associated with senescence and matrix catabolism. Autophagy is tightly suppressed by mTORC1 as well. The regulation of mTORC1 is mediated by the upstream class-I PI3K, with Akt serving as a crucial pro-survival mediator that prevents apoptosis. Furthermore, the negative feedback loop between p70/S6K and the class-I PI3K exists. To analyze the cascade-dependent functions of PI3K/Akt/mTOR signaling, gene suppression was performed using both siRNA-mediated RNAi-based and CRISPR–Cas9-based methods to target *mTOR* for both mTORC1 and mTORC2, *RAPTOR* for mTORC1, and *RICTOR* for mTORC2.

The mTOR protein is a serine/threonine kinase that integrates the major regulatory inputs of nutrients, growth factors, energy, and stress to regulate cell growth and division [33]. The mTOR exists in the following two complexes: mTOR complex 1 (mTORC1), which contains the regulatory associated protein of mTOR (RAPTOR), and mTOR complex 2 (mTORC2), which contains the rapamycin-insensitive companion of mTOR (RICTOR) [33]. The downstream effectors of mTORC1, including p70/ribosomal S6 kinase (p70/S6K), can regulate cell proliferation, viability, messenger RNA (mRNA) translation, and protein synthesis [33]. The mTORC1 is regulated by upstream Akt, an essential pro-survival mediator, by suppressing apoptosis [34]. Although the role of mTORC2 is not fully clear, mTORC2 is known to regulate Akt [34]. Then, there is the negative feedback loop between p70/S6K and the class-I PI3K [35]. Collectively, the PI3K/Akt/mTOR signaling axis essentially serves for the maintenance of healthy human disk cell, extracellular matrix, and tissue homeostasis (Figure 1) [36].

RNA interference (RNAi) is a commonly used approach for experimentally examining the function of a target gene [37]. Small interfering RNAs (siRNAs) and short hairpin RNAs (shRNAs) are the gene-silencing tools most frequently used for RNAi. The siRNAs are short double-stranded RNA molecules that target the mRNA molecules of a specific gene in the cytoplasm when transfected into cells. They support transient gene silencing because they eventually become diluted as the cells divide, which limits their use for long-term studies. However, shRNAs are introduced via DNA vectors and can integrate into the host cell genome, allowing for the stable and continuous production of hairpin RNA molecules for long-lasting gene suppression [38]. Despite the stability of shRNAs, viral vectors, such as retroviral and adenoviral vectors, have raised safety concerns because of their associated risks of insertional mutagenesis and immunogenicity, respectively [39,40]. Non-viral methods, while safer and simpler, face the challenge of lower transfection efficiency compared with viral vectors. Although RNAi technology can be used to knockdown protein expression for loss-of-function screening, the inherent incompleteness of protein depletion, temporary inhibition of gene function, and unpredictable off-target effects considerably limit its utility [41]. More recently, the clustered regularly interspaced short palindromic repeat (CRISPR)–CRISPR-associated protein 9 (Cas9) nuclease system, a third-generation engineered nuclease, has been reported to be a fast and efficient tool for conducting genome editing [42,43]. Unlike RNAi, which temporarily suppresses gene expression by degrading mRNA molecules, CRISPR–Cas9 can permanently knock out genes by directly editing the DNA, providing more robust and complete gene suppression. Additionally, CRISPR–Cas9 can target non-coding regions of DNA, while RNAi cannot. However, CRISPR–Cas9 also has some challenges. For example, off-target effects can still cause undesirable mutations. Furthermore, the DNA double-strand breaks induced by the system may trigger cellular damage responses, potentially leading to cytotoxicity. There are few reports on the use of CRISPR–Cas9 for intervertebral disk degeneration, and the effectiveness of gene-silencing approaches for disk cell therapy remains limited [44].

An mTORC1 inhibitor of rapamycin can extend the mammalian lifespan [45] and provides protective effects on human chondrocytes [46]. However, the specific inhibitory roles of PI3K/Akt/mTOR signaling in disk cells remain poorly understood. Furthermore, it is unclear which specific subunit(s) of the PI3K/Akt/mTOR signaling pathway contributes to beneficial effects observed in disk or cartilage cells. Hence, we previously performed an in vitro study of human disk cells by using siRNA-mediated RNAi, clarifying the anti-inflammatory protective advantages of RAPTOR/mTORC1 suppression by the lower cascade, and the disadvantages of mTOR/mTORC1 and mTORC2, and RICTOR/mTORC2 suppression from the upper cascade [47]. These findings were further confirmed pharmacologically in human [48] and rabbit cells [49]. However, there are still issues to be addressed, such as the off-target effects of gene silencing and pharmacological treatment. Therefore, we further designed the present in vitro study to clarify the effective gene-silencing therapeutic approaches targeting PI3K/Akt/mTOR signaling in degenerative disk cells, specifically by comparing the results using siRNA-mediated RNAi with those using CRISPR–Cas9. We hypothesized that the CRISPR–Cas9 system would have stronger gene repression effects than RNAi and provide more robust results for identifying the optimal therapeutic target component of the disk intracellular PI3K/Akt/mTOR signaling pathway.

## 2. Materials and Methods

### 2.1. Ethics Statement

All of the experimental procedures were performed under the approval and guidance of the Institutional Review Board (160004, 16 May 2016; B210176, 27 September 2021) at the Kobe University Graduate School of Medicine. Written informed consent was obtained from each patient in accordance with the principles of the Declaration of Helsinki and the laws and regulations of Japan.

### 2.2. Antibodies and Reagents

The antibodies and reagents used are listed in Table S1.

### 2.3. Cells

Human intervertebral disk NP cells were surgically obtained from patients who underwent lumbar discectomy or interbody fusion surgery for degenerative disease (*n* = 42: age, 61.6 ± 15.5 (range, 32–83) years; 23 men and 19 women). The Pfirrmann disk degeneration grade [50] was 3.7 ± 0.4 (range, 3–4), as disks with the grades 3–4 were collected to reduce the variation based on the severity. Immediately following surgery, human disk NP tissues were carefully obtained from discarded surgical waste and enzymatically digested in 1% penicillin/streptomycin-supplemented Dulbecco’s Modified Eagle’s Medium (DMEM) with 10% fetal bovine serum (FBS) and 0.114% collagenase type 2 for 1 h at 37 °C. The isolated cells were cultured as a monolayer and grown to ~80% confluence in DMEM with 10% FBS at 37 °C under 2% O_2_ to mimic the physiologically hypoxic environment of the disk [5]. To preserve their phenotype, only first-passage cells were utilized for the experiments. The cell densities used for experiments were as follows: 1.5 × 10^5^ cells per well in a 6-well plate for protein extraction, 1.2 × 10^4^ cells per well in an 8-well chamber for staining, and 5.0 × 10^3^ cells per well in a 96-well plate for counting and viability.

**Figure 2:**
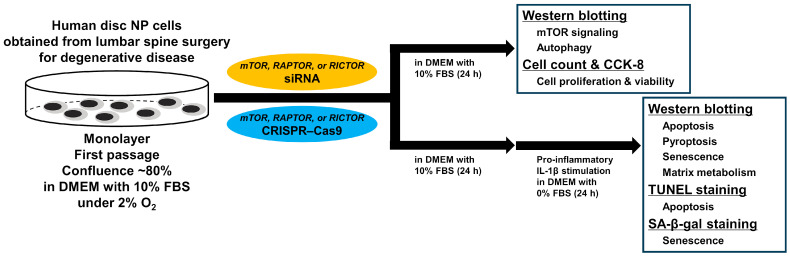
Schematic illustration of the in vitro study design. Human degenerative intervertebral disk NP cells were surgically collected from patients who underwent lumbar discectomy or interbody fusion surgery. To retain the phenotype and replicate the physiologically hypoxic intervertebral disk environment, first-passage cells were cultured under 2% O_2_ until they reached ~80% confluence. Gene knockdown and knockout targeting *mTOR*, *RAPTOR*, and *RICTOR* were performed using both siRNA-mediated RNAi and CRISPR–Cas9, respectively. After the cells were transfected for 24 h, the suppression of mTOR, RAPTOR, and RICTOR and autophagy were evaluated by Western blotting. The cell number was counted. Cell viability was measured using the CCK-8 assay to evaluate the toxicity associated with RNAi and CRISPR–Cas9. Additionally, to mimic the clinically relevant low-nutrient and inflammatory disease conditions, following siRNA or CRISPR–Cas9 treatment for 24 h, the cells were stimulated with pro-inflammatory IL-1β in serum-free DMEM for an additional 24 h. Subsequent analyses included evaluating the apoptosis, pyroptosis, senescence, and matrix metabolism using Western blotting, TUNEL staining for apoptosis, and SA-β-gal staining for senescence.

RNAi or CRISPR–Cas9 targeting *mTOR*, *RAPTOR*, or *RICTOR* was used to treat the cells. Subsequently, cells treated for 24 h in DMEM with 10% FBS were collected for protein extraction and counting, and viability assays. Western blot analysis for PI3K/Akt/mTOR signaling and autophagy was performed to confirm target gene knockdown/knockout and related changes in the pathway. The cell number was counted. Cell viability was measured using the Cell Counting Kit-8 (CCK-8) assay to evaluate the toxicity associated with RNAi and CRISPR–Cas9. To model the clinically relevant low-nutrient and inflammatory disease conditions [51], cells subjected to gene silencing for 24 h were further treated with a pro-inflammatory cytokine of IL-1β at 10 ng/mL, chosen as an effective yet non-toxic dose [29], in serum-free DMEM with 0% FBS. A serum-free medium was utilized to enable the analysis of the released proteins. Following an additional 24-h period of nutrient deprivation and inflammation, cells and supernatants were harvested for the protein extraction and staining assays. Western blot analysis for proteins related to apoptosis, pyroptosis, senescence, and matrix metabolism, apoptotic terminal deoxynucleotidyl transferase dUTP nick end-labeling (TUNEL) staining, and senescence-associated beta-galactosidase (SA-β-gal) staining were then conducted to clarify the therapeutic effects of these gene-silencing approaches (Figure 2).

### 2.4. RNAi

The specific gene knockdown of PI3K/Akt/mTOR signaling components using siRNA-mediated RNAi was applied to human disk NP cells (biological *n* = 6 per experiment). The siRNA against *mTOR* targeting mTORC1 and mTORC2, against *RAPTOR* targeting mTORC1, or against *RICTOR* targeting mTORC2 was delivered by lipofection using the reverse transfection technique, which allows for a high efficiency [52]. A non-targeting siRNA served as the negative control. To rule out off-target effects, consistent results for each target gene were verified using two distinct siRNAs with different sequences. The siRNA sequences are detailed in Table S2. Specifically, cells cultured in DMEM with 10% FBS were added to Opti-minimal essential medium I with Lipofectamine^TM^ RNAiMAX and the siRNA; then, they were incubated for 24 h. Each siRNA was applied at 60 pmol per well in a 6-well plate, 4.8 pmol per well in an 8-well chamber plate, and 2 pmol per well in a 96-well plate.

### 2.5. CRISPR–Cas9

The specific gene knockout of PI3K/Akt/mTOR signaling components using CRISPR–Cas9 was applied to human disk NP cells (biological *n* = 6, the same batches as the RNAi). The CRISPR–Cas9 knockout plasmid for *mTOR*, *RAPTOR*, or *RICTOR* was delivered by lipofection using the reverse transfection method. The knockout plasmid contained the corresponding 20-nt guide RNA. A non-targeting CRISPR–Cas9 construct was used as the negative control. To minimize off-target effects, as well as to verify the consistency, three guide RNAs with distinct sequences were used for each target gene. The guide RNA sequences are presented in Table S3. Specifically, cells cultured in DMEM with 10% FBS were transfected using jetOPTIMUS^®^ reagent, jetOPTIMUS^®^ buffer, and the CRISPR–Cas9 knockout plasmid; then, they were incubated for 24 h. The plasmid DNA was introduced at 151 pmol per well in a 6-well plate, 12 pmol per well in an 8-well chamber plate, and 9.8 pmol per well in a 96-well plate. The CRISPR–Cas9 plasmids included green fluorescent protein (GFP), allowing for the successful transfection to be visually confirmed by detecting GFP using fluorescent microscopy. The percentage of CRISPR–Cas9 plasmid-positive cells was calculated relative to the total number of 4′,6-diamidino-2-phenylindole (DAPI)-positive cells in duplicated five random low-power fields (100×) using the BZ-X700 microscope with the ImageJ software (https://imagej.net/, accessed on 1 October 2024).

### 2.6. Cell Count and Viability Assay

Cells were analyzed 24 h post-transfection to evaluate the potential toxicity of RNAi or CRISPR–Cas9. The cell number was measured by counting the adhered cells in images of duplicated five randomly selected low-power fields (100×) with a microscope using ImageJ. Cell viability was assessed by the CCK-8 dehydrogenase activity, with the absorbance values at 450 nm measured using the Model 680 microplate reader. The cell number and viability were calculated as the percentage of RNAi and CRISPR–Cas9 relative to the non-targeting control.

### 2.7. Western Blotting

Cells were lysed on ice using a 3-(*N*-morpholino)propanesulfonic acid buffer containing protease and phosphatase inhibitors. After centrifugation at 20,000× *g* for 15 min at 4 °C, the supernatant containing soluble proteins was collected. Culture media without serum were also collected and centrifuged at 1000× *g* for 10 min at 4 °C to eliminate cellular debris, and then concentrated using Amicon Ultra spin columns. The protein concentration was measured by the bicinchoninic acid assay. Samples were stored at −80 °C.

Equal 30-µg amounts of protein samples were mixed with an electrophoresis sample buffer, boiled for 5 min, and resolved on a 7.5–15.0% polyacrylamide gel. Separated proteins in a tris(hydroxymethyl)aminomethane–glycine–sodium dodecyl sulfate buffer system were electrically transblotted to a polyvinylidene difluoride membrane and probed with primary antibodies for 12 h at 4 °C (at a 1:200–1:1000 dilution), followed by incubation with secondary antibodies (at a 1:2000 dilution) for 1 h at room temperature. Positive signals were detected using enhanced chemiluminescence and visualized with the Chemilumino analyzer LAS-3000 mini. Band intensities were quantified using ImageJ. While the target protein expression levels were normalized to loading control tubulin protein expression levels, the data of the RNAi and CRISPR–Cas9 were further shown as the relative percentage of the non-targeting control or time-course sample at 0 h (100.0%).

Western blotting was performed to analyze the intracellular expression of disk NP notochord-related brachyury and CD24 [53], PI3K/Akt/mTOR signaling-related mTOR, RAPTOR, RICTOR, Akt, phosphorylated Akt, p70/S6K, and phosphorylated p70/S6K (all with a 1:1000 dilution) [33]; autophagy-related light chain 3 (LC3) and p62/sequestosome 1 (p62/SQSTM1) (both with a 1:1000 dilution) [54]; apoptosis-related poly (ADP-ribose) polymerase (PARP), cleaved PARP, and cleaved caspase-9 (all with a 1:1000 dilution) [55,56]; senescence-related p16/INK4A, p21/WAF1/CIP1, and p53 (all with a 1:1000 dilution) [57]; pyroptosis-related caspase-1, cleaved caspase-1, GSDMD, and N-terminal GSDMD (all with a 1:1000 dilution) [19,58]; matrix anabolism-related aggrecan and collagen type II alpha 1 (COL2A1) (both with a 1:1000 dilution), and loading control tubulin (with a 1:1000 dilution) in the total cell protein extracts. Western blotting was also conducted to analyze the expression levels of the released proteins, including matrix catabolism-related MMP-3 and MMP-13 (both with a 1:1000 dilution), and anti-matrix catabolism-related TIMP-1 (at a 1:200 dilution) and TIMP-2 (at a 1:1000 dilution) in the supernatant protein extracts.

### 2.8. TUNEL Staining

Cells were fixed with 4% paraformaldehyde for 10 min and subjected to fluorescein-labeled TUNEL staining for apoptotic fragmented DNA detection and DAPI for nuclear counterstaining [59]. The percentage of TUNEL-positive cells was calculated relative to the total number of DAPI-positive cells in duplicated five random low-power fields (100×) using a microscope and ImageJ.

### 2.9. SA-β-gal Staining

Senescent cells were colorimetrically detected by SA-β-gal staining at a pH of 6.0 [60]. The proportion of SA-β-gal-positive cells relative to the total cells was determined in duplicated five random low-power fields (100×) using a microscope and ImageJ.

### 2.10. Statistical Analysis

Data are presented as the mean ± standard deviation (range, minimum–maximum) in the text and as a box plot in the graphs. The cells were analyzed in duplicate. A single value was obtained by averaging the data of two technical replicates. Each experiment was conducted six times using six different patient samples (biological *n* = 6), except for the time-course analysis because of the difficulty associated with acquiring a large number of cells from surgical specimens (biological *n* = 5). Assuming normality, the paired *t*-test (the comparison between two groups at a time point) or one-way repeated measures analysis of variance (ANOVA) with the Tukey–Kramer post hoc test (comparison between ≥3 groups at a time point) was used to assess the CRISPR–Cas9 GFP positivity, cell number, cell viability, Western blot data at 24 h, TUNEL staining, and SA-β-gal staining. Then, two-way repeated measures ANOVA with the Tukey–Kramer post hoc test (comparison between ≥3 groups at multiple time points) was applied to the time-course Western blot data for up to 168 h. Statistical analysis was performed using IBM SPSS Statistics 23.0 (IBM, Armonk, NY, USA), with *p*-values of less than 0.05 considered statistically significant.

## 3. Results

### 3.1. RNAi and CRISPR–Cas9 Enhance the Selective Suppression of mTOR, RAPTOR, and RICTOR in Human Disk NP Cells

**Figure 3:**
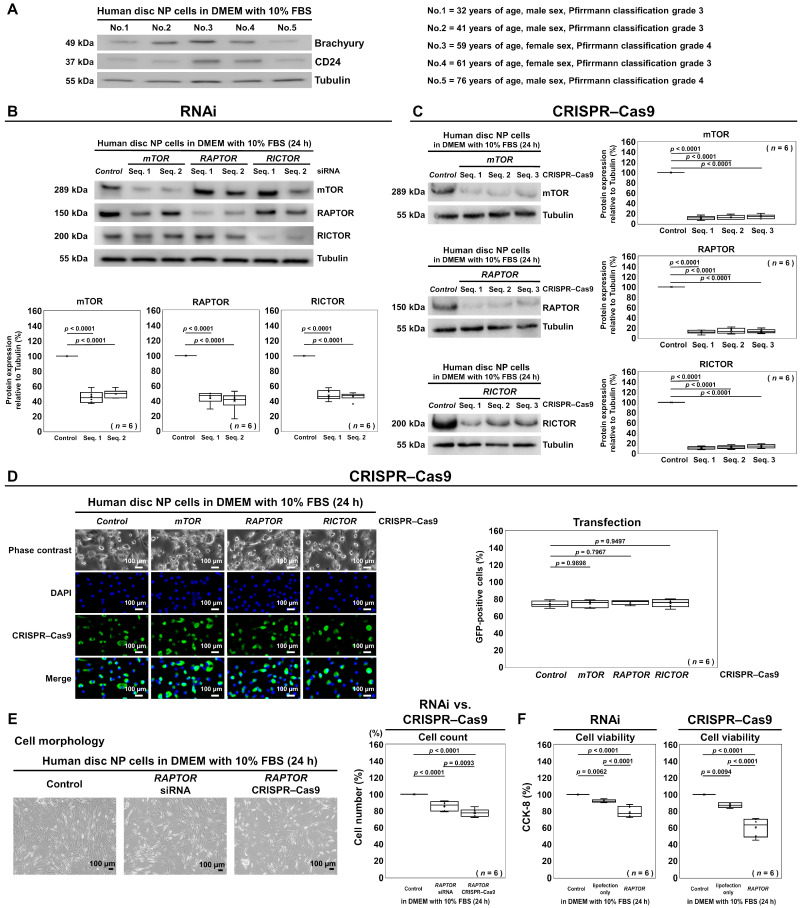
RNAi and CRISPR–Cas9 enhance the selective suppression of mTOR, RAPTOR, and RICTOR in human disk NP cells. (**A**) Western blot analysis for brachyury, CD24, and tubulin in the total protein extracts from five different batches of human disk NP cells in DMEM with 10% FBS. (**B**) Western blot analysis for mTOR, RAPTOR, RICTOR, and tubulin in the total protein extracts of human disk NP cells 24 h post-transfection with *mTOR*, *RAPTOR*, *RICTOR*, or control siRNA with each of two different sequences (Seq. 1 and Seq. 2) in DMEM with 10% FBS to assess the expression levels of the target protein relative to tubulin. (**C**) Western blot analysis for mTOR, RAPTOR, RICTOR, and tubulin in the total protein extracts of human disk NP cells 24 h after transfection with *mTOR*, *RAPTOR*, *RICTOR*, or control CRISPR–Cas9 plasmid with each of the three different guide RNA sequences (Seq. 1, Seq. 2, and Seq. 3) in DMEM with 10% FBS to assess the expression levels of the target protein relative to tubulin. (**D**) Fluorescence for phase contrast (gray), GFP (green), DAPI (blue), and merged signals in human disk NP cells 24 h post-transfection with *mTOR*, *RAPTOR*, *RICTOR*, or control siRNA containing a GFP sequence in DMEM with 10% FBS to assess the transfection efficiency of the GFP-positive cells relative to the total DAPI-positive cells. (**E**) Morphological appearance of human disk NP cells 24 h post-transfection with *RAPTOR* siRNA or *RAPTOR* CRISPR–Cas9 plasmid in DMEM with 10% FBS to assess the number of adherent cells treated relative to the control. (**F**) CCK-8 assay in human disk NP cells 24 h post-transfection with control siRNA, control CRISPR–Cas9 plasmid, lipofection only, *RAPTOR* siRNA, or *RAPTOR* CRISPR–Cas9 plasmid in DMEM with 10% FBS to assess the viability of the cells treated relative to the control. Cells were counted in duplicated five random low-power fields (100×). Statistical analysis was performed using one-way repeated measures ANOVA with the Tukey–Kramer post hoc test. Data are presented with dot and box plots (*n* = 6). In (**A**), the immunoblots shown are all results from experiments with similar outcomes (*n* = 5). In (**B**–**E**), the immunoblots and cellular images shown represent typical results from the experiments with similar outcomes (*n* = 6).

First, we confirmed the maintained phenotype of human disk NP cells originating from the notochord, showing a high specificity of brachyury and CD24 [53]. Western blotting presented consistent brachyury and CD24 expression in samples with a variation in the patient age, sex, and disk degeneration grade (Figure 3A).

Then, the selective suppression of the mTOR, RAPTOR, and RICTOR proteins in human disk cells was confirmed using both RNAi knockdown and CRISPR–Cas9 knockout methods. To identify the sequences that resulted in more efficient suppression, two different siRNA sequences were tested for each target, while three different guide RNA sequences were used for CRISPR–Cas9. The protein suppression efficiencies of the siRNA sequences were as follows: mTOR sequence no. 1, 45.6% ± 7.9%; mTOR sequence no. 2, 49.9% ± 5.3%; RAPTOR sequence no. 1, 44.4% ± 7.8%; RAPTOR sequence no. 2, 39.7% ± 12.4%; RICTOR sequence no. 1, 47.5% ± 6.6%; RICTOR sequence no. 2, 46.2% ± 5.1% (all *p* < 0.0001) (Figure 3B). The protein suppression efficiencies of the CRISPR–Cas9 guide RNA sequences were as follows: mTOR sequence no. 1, 11.1% ± 3.7%; mTOR sequence no. 2, 12.5% ± 4.0%; mTOR sequence no. 3, 13.8% ± 4.0%; RAPTOR sequence no. 1, 11.9% ± 3.4%; RAPTOR sequence no. 2, 14.0% ± 5.3%; RAPTOR sequence no. 3, 13.5% ± 3.9%; RICTOR sequence no. 1, 10.7% ± 2.8%; RICTOR sequence no. 2, 12.3% ± 3.4%; RICTOR sequence no. 3, 14.1% ± 3.7% (all *p* < 0.0001) (Figure 3C). Using these results, *mTOR* siRNA sequence no. 1, *RAPTOR* siRNA sequence no. 2, and *RICTOR* siRNA sequence no. 2 were selected for the subsequent experiments. For the CRISPR–Cas9 guide RNAs, the respective sequence no. 1 was used for *mTOR*, *RAPTOR*, and *RICTOR*.

Following the sequence selection for analysis, the presence of the GFP signal observed using fluorescence microscopy visually confirmed the successful transfection for all CRISPR–Cas9 plasmids in the cells after the 24-h treatment: control, 74.0% ± 3.6%; *mTOR*, 74.7% ± 4.2%; *RAPTOR*, 76.0% ± 2.5%; *RICTOR*, 75.2% ± 4.5% (*p* = 0.7967–0.9898) (Figure 3D).

We then examined the morphological appearance of the human disk NP cells after transfection with the *RAPTOR* siRNA or *RAPTOR* CRISPR–Cas9 plasmid. In the cells transfected with *RAPTOR* CRISPR–Cas9, a slightly decreased cell number, as well as signs of cell aggregation and detachment, were observed particularly after the RNAi and CRISPR–Cas9 interventions; for the cell counts, *RAPTOR* CRISPR–Cas9 showed a significantly greater decrease in the cell number compared with the siRNA (77.7% versus 86.0%, *p* = 0.0093) (Figure 3E). The CCK-8 assay indicated a significant reduction in the percentage of viable cells 24 h after *RAPTOR* siRNA transfection (78.8%, *p* < 0.0001). A slight decrease in viability was also noted when the cells were treated with lipofectamine alone, without any siRNA (92.0%, *p* = 0.0062). Following *RAPTOR* CRISPR–Cas9 transfection, the percentage of total cell viability decreased even further (60.5%, *p* < 0.0001). Similarly, a mild decrease was observed with lipofectamine alone (87.0%, *p* = 0.0094) (Figure 3F). In these cell count and CCK-8 assays, the introduction of the *RAPTOR* siRNA and *RAPTOR* CRISPR–Cas9 plasmid both led to a marked reduction in cell viability, although this difference was less pronounced in the cell number. Therefore, the observed additional decrease in viability by *RAPTOR* CRISPR–Cas9 compared with *RAPTOR* RNAi could result from not only greater treatment toxicity but also more robust *RAPTOR* suppression-mediated mTORC1 inhibitory effects.

### 3.2. Selective Suppression of RAPTOR/mTORC1 Inhibits Autophagy and p70/S6K but Differentially Induces Akt Activation in Human Disk NP Cells

**Figure 4:**
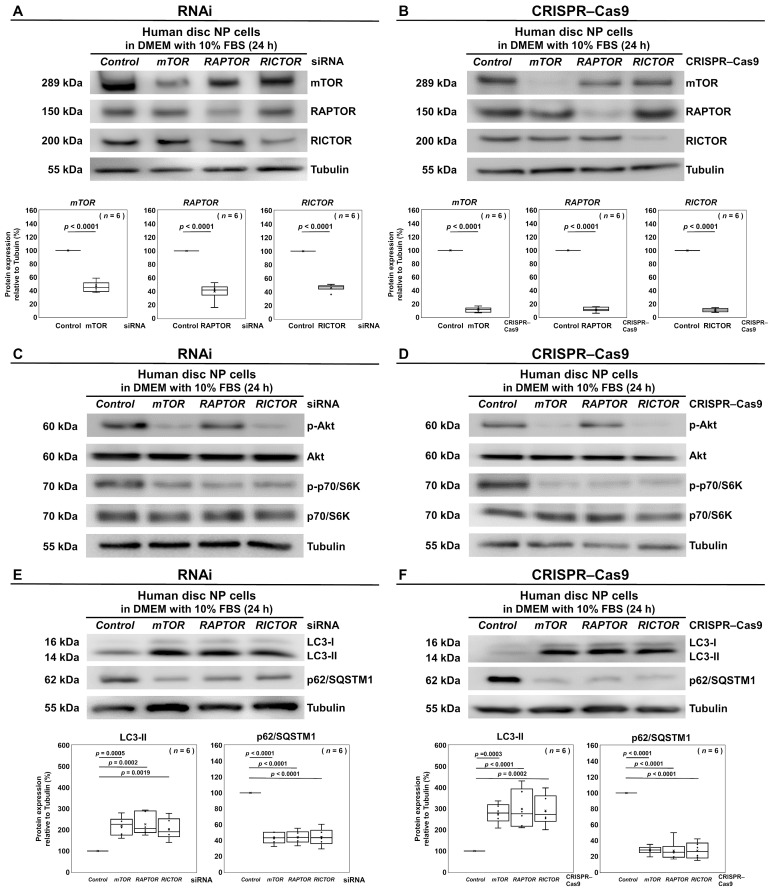
Selective suppression of RAPTOR/mTORC1 inhibits autophagy and p70/S6K but differentially induces Akt activation in human disk NP cells. (**A**) Western blot analysis for mTOR, RAPTOR, RICTOR, and tubulin in total protein extracts of human disk NP cells 24 h post-transfection with *mTOR*, *RAPTOR*, *RICTOR*, or control siRNA with the sequence showing the highest suppression efficiency in DMEM with 10% FBS to assess the expression levels of the target protein relative to tubulin. (**B**) Western blot analysis for mTOR, RAPTOR, RICTOR, and tubulin in total protein extracts of human disk NP cells 24 h after transfection with *mTOR*, *RAPTOR*, *RICTOR*, or control CRISPR–Cas9 plasmid with the sequence presenting the highest suppression efficiency in DMEM with 10% FBS to assess the expression levels of the target protein relative to tubulin. (**C**) Western blot analysis for Akt, phosphorylated Akt (p-Akt), p70/S6K, phosphorylated p70/S6K (p-p70/S6K), and tubulin in the total protein extracts of human disk NP cells 24 h post-transfection with *mTOR*, *RAPTOR*, *RICTOR*, or control siRNA in DMEM with 10% FBS. (**D**) Western blot analysis for Akt, p-Akt, p70/S6K, p-p70/S6K, and tubulin in the total protein extracts of human disk NP cells 24 h post-transfection with *mTOR*, *RAPTOR*, *RICTOR*, or control CRISPR–Cas9 plasmid in DMEM with 10% FBS. (**E**) Western blot analysis for LC3, p62/SQSTM1, and tubulin in the total protein extracts of human disk NP cells 24 h post-transfection with *mTOR*, *RAPTOR*, *RICTOR*, or control siRNA in DMEM with 10% FBS to assess the expression levels of the target protein relative to tubulin. (**F**) Western blot analysis for LC3, p62/SQSTM1, and tubulin in the total protein extracts of human disk NP cells 24 h after transfection with *mTOR*, *RAPTOR*, *RICTOR*, or control CRISPR–Cas9 plasmid in DMEM with 10% FBS to assess the expression levels of the target protein relative to tubulin. Statistical analysis was performed using the paired *t*-test or one-way repeated measures ANOVA with the Tukey–Kramer post hoc test. Data are presented with dot and box plots (*n* = 6). The immunoblots shown represent the typical results from experiments with similar outcomes (*n* = 6).

We next evaluated how the RNAi and CRISPR–Cas9 editing affected the PI3K/Akt/mTOR signaling pathway in human disk cells. First, we confirmed again the efficacy and specificity of the selected siRNA and CRISPR–Cas9 guide RNA sequences, presenting 53.8–60.3% protein suppression by RNAi and 88.1–89.3% by CRISPR–Cas9 targeting *mTOR*, *RAPTOR*, and *RICTOR* (all *p* < 0.0001) (Figure 4A,B). Then, Western blot analysis demonstrated that the *mTOR* and *RICTOR* siRNAs reduced the phosphorylation levels of p70/S6K and Akt, while the *RAPTOR* siRNA specifically decreased p70/S6K but maintained or increased the Akt phosphorylation levels (Figure 4C). Similar but further distinct trends were observed with CRISPR–Cas9 (Figure 4D). These findings indicate the consistent inhibition of downstream PI3K/Akt/mTOR effectors, including p70/S6K, and differential upstream Akt induction, the degree of which is more remarkable by CRISPR–Cas9 than by RNAi.

We subsequently evaluated the effects of the selective inhibition of the PI3K/Akt/mTOR signaling pathway by RNAi and CRISPR–Cas9 on disk cellular autophagy. Western blotting revealed that all of the siRNA treatments led to increased LC3-II and decreased p62/SQSTM1 protein expression levels (Figure 4E). Then, the CRISPR–Cas9 treatments exhibited more pronounced effects compared with the siRNAs (Figure 4F). The microtubule-associated protein 1 LC3 is a ubiquitin-like protein, present in the cytosolic LC3-I or phosphatidylethanolamine-conjugated LC3-II form [61]. The LC3-II is specifically associated with completed autophagosomes, indicating a positive correlation with autophagy [61]. The p62/SQSTM1 and polyubiquitinated proteins bound to it are sequestered into completed autophagosomes and subsequently degraded within autolysosomes, reflecting a negative correlation with autophagic activity [54]. Therefore, the observed findings clearly support enhanced autophagy [54], which is also consistent under the treatment of CRISPR–Cas9 compared with RNAi.

### 3.3. Selective Suppression of RAPTOR/mTORC1 Inhibits Apoptosis in Human Disk NP Cells

**Figure 5:**
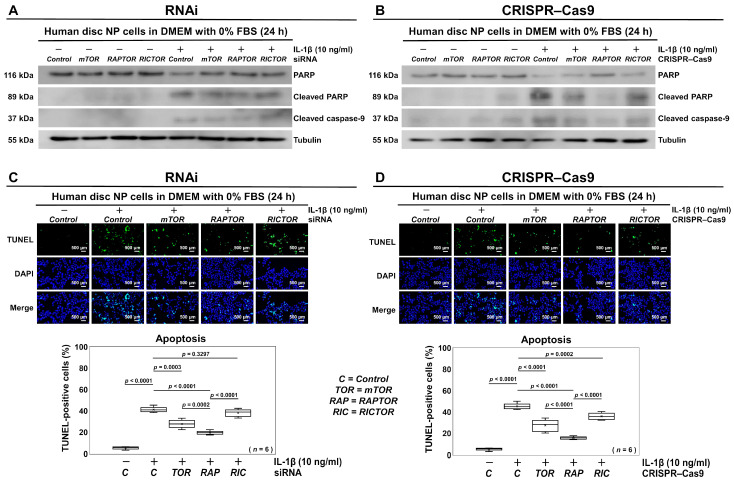
Selective suppression of RAPTOR/mTORC1 inhibits apoptosis in human disk NP cells. (**A**) Western blot analysis for PARP, cleaved PARP, cleaved caspase-9, and tubulin in the total protein extracts of human disk NP cells 24 h post-transfection with *mTOR*, *RAPTOR*, *RICTOR*, or control siRNA in 10 ng/mL IL-1β-supplemented DMEM with 0% FBS. (**B**) Western blot analysis for PARP, cleaved PARP, cleaved caspase-9, and tubulin in the total protein extracts of human disk NP cells 24 h after transfection with *mTOR*, *RAPTOR*, *RICTOR*, or control CRISPR–Cas9 plasmid in 10 ng/mL IL-1β-supplemented DMEM with 0% FBS. (**C**) Fluorescence for TUNEL (green), DAPI (blue), and merged signals in human disk NP cells 24 h post-transfection with *mTOR*, *RAPTOR*, *RICTOR*, or control siRNA in 10 ng/mL IL-1β-supplemented DMEM with 0% FBS to assess the ratio of TUNEL-positive cells relative to the total DAPI-positive cells. (**D**) Fluorescence for TUNEL (green), DAPI (blue), and merged signals in human disk NP cells 24 h after transfection with *mTOR*, *RAPTOR*, *RICTOR*, or control CRISPR–Cas9 plasmid in 10 ng/mL IL-1β-supplemented DMEM with 0% FBS to assess the ratio of TUNEL-positive cells relative to the total DAPI-positive cells. Cells were counted in duplicated five random low-power fields (100×). Statistical analysis was performed using one-way repeated measures ANOVA with the Tukey–Kramer post hoc test. Data are presented with dot and box plots (*n* = 6). The immunoblots and cellular images shown represent typical results from the experiments with similar outcomes (*n* = 6).

We evaluated the effects of the selective inhibition of PI3K/Akt/mTOR signaling by RNAi and CRISPR–Cas9 on disk cell apoptosis. Apoptosis was assessed using Western blotting and TUNEL staining. To mimic the clinically relevant disease conditions, the cells were treated with 10 ng/mL IL-1β for an additional 24 h, after which the medium was replaced with serum-free DMEM following the RNAi or CRISPR–Cas9 treatment. The applied concentration of IL-1β was selected as an effective but non-toxic concentration based on a dose-dependent reduction in the cell viability, as previously reported [29]. Western blot analysis revealed that the effects of the *mTOR*, *RAPTOR*, and *RICTOR* siRNA transfections inhibited the reduced PARP, increased the cleaved PARP, and increased the cleaved caspase-9 protein expression levels, all of which suggest mitochondrial apoptosis (Figure 5A). Importantly, the CRISPR–Cas9 treatment, particularly with the plasmid targeting *RAPTOR*, showed stronger inhibitory effects than RNAi (Figure 5B).

The percentage of apoptotic TUNEL-positive cells, 41.2%, increased under the inflammatory condition with the supplementation of IL-1β compared to 5.3% without IL-1β (*p* < 0.0001). However, this increase was significantly decreased by *RAPTOR* siRNA transfection, at 19.7% (*p* < 0.0001) (Figure 5C), with a similar significant reduction observed with *RAPTOR* CRISPR–Cas9 (*p* < 0.0001) (Figure 5D). Integrated with both the RNAi and CRISPR–Cas9 findings, the selective suppression of RAPTOR/mTORC1 appears to inhibit apoptosis, the most common form of programmed cell death.

### 3.4. Selective Suppression of RAPTOR/mTORC1 Inhibits Pyroptosis in Human Disk NP Cells

**Figure 6:**
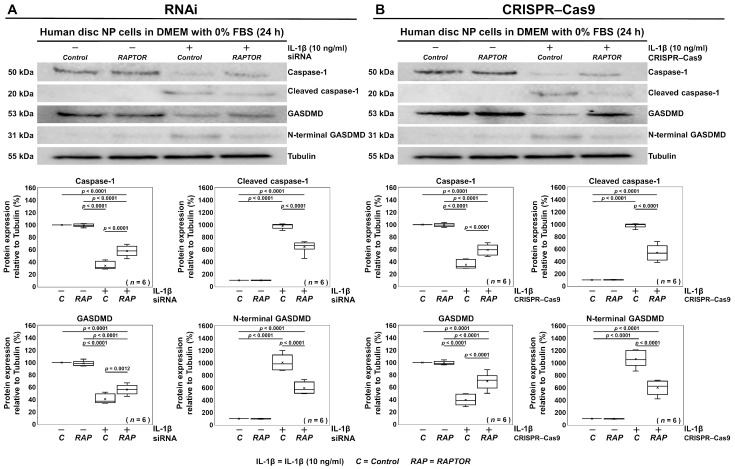
Selective suppression of RAPTOR/mTORC1 inhibits pyroptosis in human disk NP cells. (**A**) Western blot analysis for caspase-1, cleaved caspase-1, GSDMD, N-terminal GSDMD, and tubulin in the total protein extracts of human disk NP cells 24 h post-transfection with *mTOR*, *RAPTOR*, *RICTOR*, or control siRNA in 10 ng/mL IL-1β-supplemented DMEM with 0% FBS to assess the expression levels of the target protein relative to tubulin. (**B**) Western blot analysis for caspase-1, cleaved caspase-1, GSDMD, N-terminal GSDMD, and tubulin in the total protein extracts of human disk NP cells 24 h post-transfection with *mTOR*, *RAPTOR*, *RICTOR*, or control CRISPR–Cas9 plasmid in 10 ng/mL IL-1β-supplemented DMEM with 0% FBS to assess the expression levels of the target protein relative to tubulin. Statistical analysis was performed using one-way repeated measures ANOVA with the Tukey–Kramer post hoc test. Data are presented with dot and box plots (*n* = 6). The immunoblots shown represent typical results from the experiments with similar outcomes (*n* = 6).

We additionally examined the impact of the *RAPTOR* siRNA and *RAPTOR* CRISPR–Cas9 treatments on disk cell pyroptosis under the same nutrition-deprived, inflammatory condition as in the apoptosis experiments. Pyroptosis, a form of programmed cell death, is strongly linked to inflammatory responses [20,21]. It is primarily driven by the activation of inflammatory caspases, especially caspase-1, which cleaves GSDMD [20,21]. This cleavage produces a pore-forming N-terminal fragment of GSDMD, which facilitates the release of pro-inflammatory cytokines and eventually leads to cell lysis [20,21]. In this study, pro-inflammatory IL-1β administration resulted in decreased caspase-1, increased cleaved caspase-1, decreased GSDMD, and increased N-terminal GSDMD protein expression levels, as indicated by the Western blot analysis. These expression patterns confirmed the induction of pyroptosis in human disk NP cells. However, these changes were mitigated by *RAPTOR* siRNA treatment (Figure 6A). Similarly, *RAPTOR* CRISPR–Cas9 also demonstrated inhibitory effects on cell pyroptosis (Figure 6B). Both the RNAi and CRISPR–Cas9 treatments targeting *RAPTOR* suggest that the selective suppression of RAPTOR/mTORC1 also inhibits pyroptosis, another form of programmed cell death.

### 3.5. Selective Suppression of RAPTOR/mTORC1 Inhibits Senescence in Human Disk NP Cells

**Figure 7:**
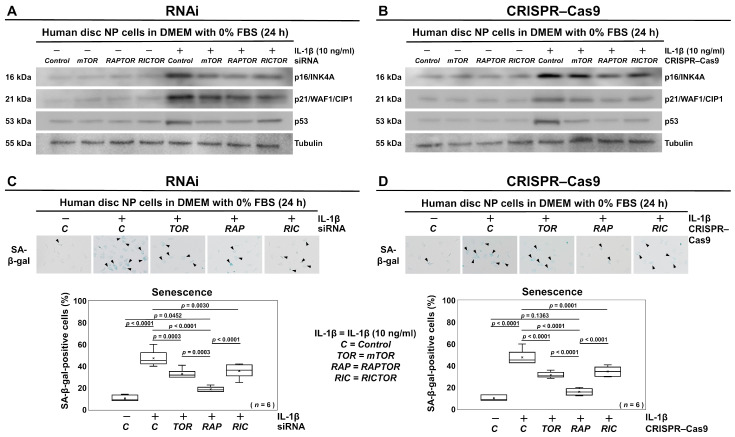
Selective suppression of RAPTOR/mTORC1 inhibits senescence in human disk NP cells. (**A**) Western blot analysis for p16/INK4A, p21/WAF1/CIP1, p53, and tubulin in the total protein extracts of human disk NP cells 24 h post-transfection with *mTOR*, *RAPTOR*, *RICTOR*, or control siRNA in 10 ng/mL IL-1β-supplemented DMEM with 0% FBS. (**B**) Western blot analysis for p16/INK4A, p21/WAF1/CIP1, p53, and tubulin in the total protein extracts of human disk NP cells 24 h after transfection with *mTOR*, *RAPTOR*, *RICTOR*, or control CRISPR–Cas9 plasmid in 10 ng/mL IL-1β-supplemented DMEM with 0% FBS. (**C**) Colorimetric assay for the SA-β-gal signals (blue, indicated by black arrowheads) in human disk NP cells 24 h post-transfection with *mTOR*, *RAPTOR*, *RICTOR*, or control siRNA in 10 ng/mL IL-1β-supplemented DMEM with 0% FBS to assess the ratio of SA-β-gal-positive cells relative to the total cells. (**D**) Colorimetric assay for the SA-β-gal signals (blue, indicated by black arrowheads) in human disk NP cells 24 h post-transfection with *mTOR*, *RAPTOR*, *RICTOR*, or control CRISPR–Cas9 plasmid in 10 ng/mL IL-1β-supplemented DMEM with 0% FBS to assess the ratio of SA-β-gal-positive cells relative to the total cells. Cells were counted in duplicated five random low-power fields (100×). Statistical analysis was performed using one-way repeated measures ANOVA with the Tukey–Kramer post hoc test. Data are presented with dot and box plots (*n* = 6). The immunoblots and cellular images shown represent typical results from the experiments with similar outcomes (*n* = 6).

We further evaluated the effects of selective PI3K/Akt/mTOR signaling pathway inhibition by RNAi and CRISPR–Cas9 on disk cellular senescence, which was assessed using Western blotting and SA-β-gal staining. We conducted the experiments under the same nutrient deprivation and inflammation as in the apoptosis and pyroptosis experiments. Western blot analysis demonstrated that the IL-1β-treatment-induced elevations in protein expression levels of the senescence markers, including p16/INK4A, p21/WAF1/CIP1, and p53, were alleviated following transfection with the *mTOR*, *RAPTOR*, or *RICTOR* siRNAs (Figure 7A). Remarkably, CRISPR–Cas9 targeting *RAPTOR* exhibited a particularly stronger inhibitory effect on senescence-related p16/INK4A, p21/WAF1/CIP1, and p53 expression (Figure 7B).

The percentage of SA-β-gal-positive cells also increased from 10.4% to 47.3% under the inflammatory condition (*p* < 0.0001). However, this increase was significantly decreased by the *RAPTOR* siRNA treatment (*p* < 0.0001) (Figure 7C), with a similar significant reduction observed with the *RAPTOR* CRISPR–Cas9 treatment (*p* < 0.0001) (Figure 7D). These findings all reflect the PI3K/Akt/mTOR signaling suppression-mediated cell cycle inhibition, particularly by *RAPTOR* CRISPR–Cas9.

### 3.6. Selective Suppression of RAPTOR/mTORC1 Increases Matrix Anabolism Through Decreased Catabolic Enzymes in Human Disk NP Cells

**Figure 8:**
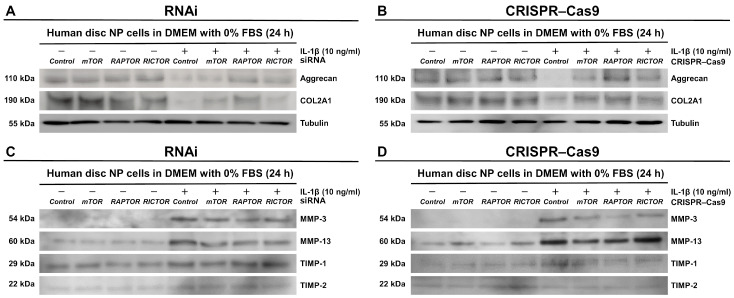
Selective suppression of RAPTOR/mTORC1 increases matrix anabolism through decreased catabolic enzymes in human disk NP cells. (**A**) Western blot analysis for aggrecan, COL2A1, and tubulin in the total protein extracts of human disk NP cells 24 h post-transfection with *mTOR*, *RAPTOR*, *RICTOR*, or control siRNA in 10 ng/mL IL-1β-supplemented DMEM with 0% FBS. (**B**) Western blot analysis for aggrecan, COL2A1, and tubulin in the total protein extracts of human disk NP cells 24 h post-transfection with *mTOR*, *RAPTOR*, *RICTOR*, or control CRISPR–Cas9 plasmid in 10 ng/mL IL-1β-supplemented DMEM with 0% FBS. (**C**) Western blot analysis for MMP-3, MMP-13, TIMP-1, and TIMP-2 in the supernatant protein extracts of human disk NP cells 24 h post-transfection with *mTOR*, *RAPTOR*, *RICTOR*, or control siRNA in 10 ng/mL IL-1β-supplemented DMEM with 0% FBS. (**D**) Western blot analysis for MMP-3, MMP-13, TIMP-1, and TIMP-2 in the supernatant protein extracts of human disk NP cells 24 h post-transfection with *mTOR*, *RAPTOR*, *RICTOR*, or control CRISPR–Cas9 plasmid in 10 ng/mL IL-1β-supplemented DMEM with 0% FBS. The immunoblots shown represent typical results from the experiments with similar outcomes (*n* = 6).

We then examined the effects of selective PI3K/Akt/mTOR signaling inhibition on the disk extracellular matrix metabolism, which was conducted under the inflammation and limited nutrition described previously. Pro-inflammatory IL-1β stimulation led to downregulated anabolic aggrecan and COL2A1 protein expression levels. These levels increased with the *RAPTOR* siRNA treatment (Figure 8A), with an even stronger effect observed with the *RAPTOR* CRISPR–Cas9 treatment (Figure 8B). In the culture supernatants, the IL-1β treatment also shifted the matrix metabolism towards catabolism, resulting in significantly elevated levels of the catabolic enzymes MMP-3 and MMP-13 relative to the anti-catabolic factors TIMP-1 and TIMP-2. The release of MMP-3 and MMP-13 was significantly decreased by all of the examined siRNAs (Figure 8C) and CRISPR–Cas9 plasmids (Figure 8D). However, the TIMP-1 and TIMP-2 levels were less affected (Figure 8C,D). These findings were consistent between RNAi and CRISPR–Cas9, especially under the treatment targeting *RAPTOR*.

### 3.7. RNAi Facilitates Transient RAPTOR Gene Knockdown but CRISPR–Cas9 Provides Extensive RAPTOR Gene Knockout in Human Disk NP Cells

**Figure 9:**
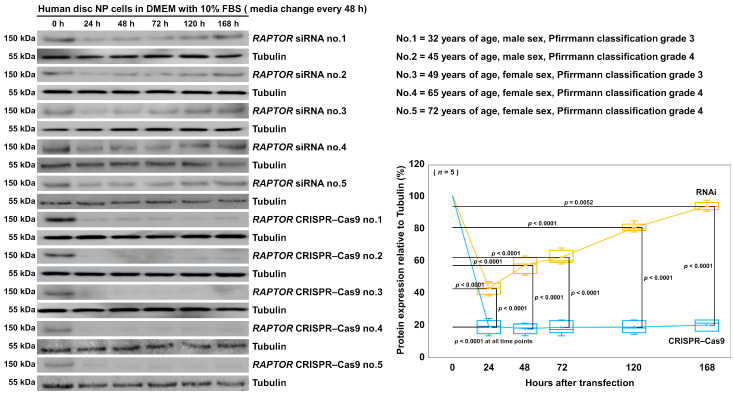
RNAi facilitates transient *RAPTOR* gene knockdown but CRISPR–Cas9 provides extensive *RAPTOR* gene knockout in human disk NP cells. Western blot analysis for RAPTOR and tubulin in the total protein extracts from five different batches of human disk NP cells at 0, 24, 48, 72, 120, and 168 h post-transfection with *RAPTOR* siRNA or CRISPR–Cas9 plasmid in 10% FBS-supplemented DMEM with a media change every 48 h to assess the time-course expression levels of the RAPTOR protein relative to tubulin. Statistical analysis was performed using two-way repeated measures ANOVA with the Tukey–Kramer post hoc test. Data are represented as the mean ± standard deviation (*n* = 5). The immunoblots shown are all results from the experiments with similar outcomes (*n* = 5).

With both the siRNA and CRISPR–Cas9 approaches, we finally examined the duration of the suppression effects using time-course experiments. The protein suppression effects of *RAPTOR* RNAi and CRISPR–Cas9 were assessed at 0, 24, 48, 72, 120, and 168 h after transfection in three different cell batches. Time-course Western blotting showed that RNAi displayed the temporary suppression of RAPTOR protein expression at 24–120 h post-transfection (*p* < 0.0001), the effect of which disappeared at 168 h post-transfection (*p* = 0.0052). In contrast, CRISPR–Cas9 exhibited the sustained suppression of the RAPTOR protein relative to the control at all of the following time points of 24 h or later (all *p* < 0.0001). At 168 h after transfection, the RAPTOR protein expression was kept suppressed to 16.8% (suppression ratio: 83.2%) by CRISPR–Cas9, but recovered to 91.2% (suppression ratio: 8.8%) by RNAi (*p* < 0.0001) (Figure 9). This clearly indicates that CRISPR–Cas9 gene knockout could maintain a higher level of protein suppression over a longer period compared with RNAi gene knockdown.

## 4. Discussion

Gene editing technologies have served as powerful tools in basic medical research for elucidating gene functions, thereby deepening our understanding of various biological processes and advancing the development of new therapies and biotechnologies. Before the advent of CRISPR–Cas9, techniques such as zinc finger nucleases (ZFNs) [62] and transcription activator-like effector nucleases (TALENs) [63] were widely used. The ZFNs involve a combination of zinc finger domains that recognize specific DNA sequences and a FokI nuclease domain that cuts the DNA. Similarly, TALENs utilize TALE domains to bind DNA, with the FokI nuclease creating double-strand breaks. While both techniques offer high specificity in gene targeting, they are complex and costly to design and produce.

After emerging in 2012, the CRISPR–Cas9 method has revolutionized the field of gene editing [42]. Derived from a bacterial immune system mechanism, CRISPR–Cas9 uses the Cas9 enzyme and guide RNA to precisely cut specific DNA sequences. The ease of designing guide RNAs and the ability to rapidly and cost-effectively target a wide range of genes have made CRISPR–Cas9 a preferred method for both research and therapeutic applications [64]. Additionally, using this approach to edit multiple genes simultaneously offers significant advantages over other techniques [65].

The siRNA-mediated RNAi method has been used for gene silencing, specifically by targeting certain mRNAs for degradation to temporarily reduce gene expression levels [66]. This technology has been instrumental in detailed studies of specific gene functions because of its simple design and application [67]. The primary differences between the RNAi and CRISPR–Cas9 methods depend on their gene suppression mechanisms and effective duration. While siRNA-mediated RNAi is used for transient gene suppression through mRNA degradation, CRISPR–Cas9 directly edits the genome’s DNA, allowing for permanent gene modifications [68]. In particular, CRISPR–Cas9 is valuable in research and therapies that require more robust and lasting gene suppression [69].

We previously used siRNA-mediated RNAi gene silencing to analyze the function of the human intervertebral disk cellular PI3K/Akt/mTOR signaling pathway, in which the selective suppression of RAPTOR/mTORC1 could enhance autophagy and prevent inflammation-induced apoptosis, senescence, and matrix catabolism, potentially paving the way for novel therapeutic approaches [47]. In the current study, we used both RNAi and CRISPR–Cas9 to examine the effects of the selective PI3K/Akt/mTOR signaling pathway inhibition in human disk NP cells. Our results confirmed that CRISPR–Cas9 achieved a higher suppression of mTOR, RAPTOR, and RICTOR proteins compared with RNAi. This led to increased autophagy and reduced apoptosis, pyroptosis, senescence, and matrix catabolism under nutrient deprivation and inflammation. Moreover, selectively inhibiting RAPTOR/mTORC1 with CRISPR–Cas9 produced stronger effects than with RNAi, likely because of the superior gene suppression capabilities of CRISPR–Cas9. This can enhance the reliability of the RAPTOR/mTORC1 functional analysis data.

In this study, despite a marked inhibition of apoptosis by RNAi and CRISPR–Cas9 targeting *RAPTOR* in human disk NP cells, the cell number and viability both declined by these gene-silencing treatments. This is assumed to result from the RAPTOR/mTORC1 suppression-mediated inhibition of p70/S6K, which is an important regulator of mRNA translation, protein synthesis, and cell proliferation [33]. The observed findings of the cell viability, monitored under a serum-supplemented condition, should reflect the total cell population as well as the individual cell metabolic activity, which also indicates the successful suppression of RAPTOR/mTORC1.

The observed effects of anti-apoptosis and anti-senescence by RNAi and CRISPR–Cas9 targeting *RAPTOR* on human disk cells are noteworthy, although limited by RNAi and CRISPR–Cas9 targeting *mTOR* and *RICTOR*. These are possibly due to increased Akt phosphorylation by RAPTOR/mTORC1 suppression, through the negative feedback loop between p70/S6K and the class-I PI3K [35]. This Akt induction does not occur by mTORC2 suppression resulting from *mTOR* and *RICTOR* gene-silencing approaches. The key function of Akt is to block apoptosis by inhibiting proapoptotic proteins, including the BCL2-associated agonist of cell death, directly or through effects on forkhead box O and p53 [34]. Then, Akt enhances cell proliferation primarily through the induction of mTORC1 and p70/S6K, and weakly through the inhibition of negative cell cycle regulators, including p21/WAF1/CIP1 and p53 [34,70]. Thus, the anti-senescence potential by PI3K/Akt/mTOR-signaling modulation largely depends on the severity of mTORC1 suppression. In fact, the administration of an allosteric Akt inhibitor, MK-2206, markedly exacerbated human disk cell apoptosis and senescence [48]. Collectively, Akt-activation-mediated anti-apoptosis and mTORC1-suppression-mediated anti-senescence suggest the selective suppression of RAPTOR as a therapeutic application for degenerative disk disease.

The role of inflammation, a response to stress, should also be discussed. We used IL-1β to simulate inflammatory conditions in human disk cells. The IL-1β is a pro-inflammatory cytokine closely associated with the pathogenesis of intervertebral disk degeneration [51], with increased IL-1β production levels correlating with higher disease severity [71]. Based on more recent comprehensive molecular expression profiles, IL-1β has driven extracellular matrix degradation, tissue remodeling, and inflammatory responses, accelerating intervertebral disk degeneration, cartilage breakdown, and osteoarthritic joint damage through a vicious cycle of cytokine production and immune cell infiltration [72,73]. By introducing IL-1β into the in vitro model, we aimed to reproduce the inflammatory microenvironment characteristic of degenerative disks. This approach could provide a relevant platform to seek the cellular mechanisms underlying disk pathology and clarify potential therapeutic interventions.

While inhibiting RAPTOR/mTORC1 using CRISPR–Cas9 shows promise as a potential therapeutic tool because of its potent protective effects on intervertebral disk cells, it also raises significant concerns. Our experiments revealed a significant cell viability decrease following CRISPR–Cas9 introduction, suggesting a potential negative impact on disk health in vivo. Furthermore, although CRISPR–Cas9 provides sustained gene suppression compared with RNAi, the long-term effects of such suppression must be carefully considered. The irreversibility of genome editing poses a risk of permanent side effects. The potential for long-term off-target effects could induce unforeseen genetic mutations, possibly resulting in cellular dysfunction or an increased risk of tumorigenesis [74]. Therefore, in this regard, the transient nature of RNAi may offer a safety advantage.

However, given the anatomical characteristics of the intervertebral disk [75,76], CRISPR–Cas9 might still hold the therapeutic potential if its safety can be ensured. The encapsulated and isolated structure of the disk suggests that localized gene therapy through a direct CRISPR–Cas9 injection could be a viable approach.

Although the CRISPR–Cas9 technology is undoubtedly a powerful tool for investigating the PI3K/Akt/mTOR signaling pathway in intervertebral disk cells, its clinical application requires careful consideration given the challenges. The potential risks associated with off-target effects, reduced cell viability, and the permanent nature of gene editing must be weighed against the benefits. As a research tool, CRISPR–Cas9 provides invaluable insights into gene function. Translating this method into safe and effective therapeutic approaches for conditions like intervertebral disk degeneration remains complex and necessitates further study and development.

## 5. Conclusions

This study compared the effects of PI3K/Akt/mTOR signaling pathway inhibition in degenerative intervertebral disk NP cells using RNAi and CRISPR–Cas9. While RNAi enhanced transient gene knockdown, CRISPR–Cas9 accomplished sustained gene knockout. The selective inhibition of RAPTOR/mTORC1 consistently led to increased autophagy and decreased apoptosis, pyroptosis, senescence, and matrix catabolism; especially, CRISPR–Cas9 exerted more pronounced effects compared to RNAi. These findings suggest that RAPTOR/mTORC1 is a promising therapeutic target for degenerative disk disease, firmly established by CRISPR–Cas9. However, the observed decrease in cell viability as well as the irreversible nature of gene editing with CRISPR–Cas9 highlight the need for careful consideration of its safety in clinical applications. Future investigations should clarify these aspects to ensure the safe and effective translation of these findings into therapeutic strategies.

## Data Availability

The data presented in this study are available on request from the corresponding author.

## Supplementary Materials

The following supporting information can be downloaded at: https://www.mdpi.com/article/10.3390/cells13232030/s1, Table S1: List of the antibodies, reagents, and instruments used; Table S2: List of siRNA sequences used; Table S3: List of CRISPR–Cas9 guide RNA sequences used.
