# Supplementary material for: Gene-Silencing Therapeutic Approaches Targeting PI3K/Akt/mTOR Signaling in Degenerative Intervertebral Disk Cells: An In Vitro Comparative Study Between RNA Interference and CRISPR–Cas9
Source: Cells. 2024 Dec 9;13(23):2030. doi: 10.3390/cells13232030 (PMC11640589; doi:10.3390/cells13232030)
Supplement: Supplementary file 1 [file cells-13-02030-s001.zip › cells-3314943-supplementary.pdf]

Table S1. List of the antibodies, reagents, and instruments used.

Product	Catalog number	Manufacturer
Antibody		
Brachyury (mouse, monoclonal)	sc-166962	Santa Cruz Biotechnology (Santa Cruz, CA)
CD24 (mouse, monoclonal)	sc-19585	Santa Cruz Biotechnology (Santa Cruz, CA)
mTOR (rabbit, monoclonal)	2983	Cell Signaling Technology (Danvers, MA)
RAPTOR (rabbit, monoclonal)	2280	Cell Signaling Technology (Danvers, MA)
RICTOR (rabbit, monoclonal)	2114	Cell Signaling Technology (Danvers, MA)
Akt (rabbit, monoclonal)	4691	Cell Signaling Technology (Danvers, MA)
Phosphorylated Akt (mouse, monoclonal)	4060	Cell Signaling Technology (Danvers, MA)
p70/S6K (mouse, monoclonal)	2708	Santa Cruz Biotechnology (Santa Cruz, CA)
Phosphorylated p70/S6K (mouse, monoclonal)	9234	Santa Cruz Biotechnology (Santa Cruz, CA)
LC3 (mouse, monoclonal)	3868	Santa Cruz Biotechnology (Santa Cruz, CA)
p62/SQSTM1 (mouse, monoclonal)	ab56416	Abcam (Cambridge, UK)
PARP (rabbit, monoclonal)	9532	Cell Signaling Technology (Danvers, MA)
Cleaved PARP (mouse, monoclonal)	5625	Cell Signaling Technology (Danvers, MA)
Cleaved caspase-9 (rabbit, polyclonal)	9501	Cell Signaling Technology (Danvers, MA)
p16/INK4a (mouse, monoclonal)	sc-1661	Santa Cruz Biotechnology (Santa Cruz, CA)
p21/WAF1/CIP1 (mouse, monoclonal)	sc-6246	Santa Cruz Biotechnology (Santa Cruz, CA)
p53 (mouse, monoclonal)	2524	Cell Signaling Technology (Danvers, MA)
Caspase-1 (rabbit, monoclonal)	24232	Cell Signaling Technology (Danvers, MA)
Cleaved caspase-1(rabbit, monoclonal)	89332	Cell Signaling Technology (Danvers, MA)
GSDMD (rabbit, monoclonal)	69469	Cell Signaling Technology (Danvers, MA)
N-terminal GASDMD (rabbit, monoclonal)	ab215203	Abcam (Cambridge, UK)
Aggrecan (mouse, monoclonal)	ab36861	Abcam (Cambridge, UK)
COL2A1 (mouse, monoclonal)	sc-52658	Santa Cruz Biotechnology (Santa Cruz, CA)
MMP-3 (rabbit, monoclonal)	ab52915	Abcam (Cambridge, UK)
MMP-13 (rabbit, polyclonal)	ab39012	Abcam (Cambridge, UK)
TIMP-1 (rabbit, polyclonal)	sc-5538	Santa Cruz Biotechnology (Santa Cruz, CA)
TIMP-2 (rabbit, monoclonal)	5738	Cell Signaling Technology (Danvers, MA)
Tubulin (mouse, monoclonal)	T9026	Sigma-Aldrich (St. Louis, MO)
RNAi		
siRNA duplexes		VectorBuilder (Chicago, IL)
Lipofectamine RNAiMAX transfection reagent	13778150	Thermo Fisher Scientific (Waltham, MA)
Opti-minimal essential medium I	3198070	Thermo Fisher Scientific (Waltham, MA)
CRISPR–Cas9		
CRISPR–Cas9 knockout plasmids		VectorBuilder (Chicago, IL)
jetOPTIMUS® reagent	101000051	Polyplus-transfection SA (Illkirch, France)
jetOPTIMUS® buffer	201000001	Polyplus-transfection SA (Illkirch, France)
Cell culture		
DMEM	D5796	Sigma-Aldrich (St. Louis, MO)
FBS	F2442	Sigma-Aldrich (St. Louis, MO)
Penicillin/streptomycin	26253-84	Nacalai Tesque (Kyoto, Japan)
Collagenase type 2	LS004176	Worthington Biochemical (Lakewood, NJ)
Human recombinant IL-1β	ALX-520-001-C010	Enzo Life Science (Farmingdale, NY)
CCK-8	CK04	Dojindo Laboratories (Kumamoto, Japan)
Model 680 microplate reader		Bio-Rad (Hercules, CA)

Product	Catalog number	Manufacturer
Staining		
In situ cell death detection kit, fluorescein	11684795910	Roche (Basel, Switzerland)
SA-β-gal staining kit	9860	Cell Signaling Technology (Danvers, MA)
4% Paraformaldehyde phosphate buffer solution	163-20145	Wako (Osaka, Japan)
DAPI	D1306	Thermo Fisher Scientific (Waltham, MA)
BZ-X700 microscope		Keyence (Osaka, Japan)
Western blotting		
3-(*N*-Morpholino)propanesulfonic acid	23438-64	Nacalai Tesque (Kyoto, Japan)
Protease inhibitor cocktail	25955-11	Nacalai Tesque (Kyoto, Japan)
Tris(hydroxymethyl)aminomethane	35434-21	Nacalai Tesque (Kyoto, Japan)
Chemi-Lumi One Super	02230-30	Nacalai Tesque (Kyoto, Japan)
Phosphatase inhibitor cocktails 2	P5726	Sigma-Aldrich (St. Louis, MO)
Phosphatase inhibitor cocktails 3	P0044	Sigma-Aldrich (St. Louis, MO)
Glycine	12-1210	Sigma-Aldrich (St. Louis, MO)
Pierce BCA protein assay kit	23227	Thermo Fisher Scientific (Waltham, MA)
7.5%–15.0% Polyacrylamide gel	SDG-581	Bio Craft (Tokyo, Japan)
4× Laemmli sample buffer	1610747	Bio-Rad (Hercules, CA)
2-Mercaptoethanol	21438-82	Nacalai Tesque (Kyoto, Japan)
SDS	191-07145	Wako (Osaka, Japan)
0.2 PVDF Western blotting membrane	10600021	GE Healthcare (Chicago, IL)
Anti-rabbit secondary antibody	NA934	GE Healthcare (Chicago, IL)
Anti-mouse secondary antibody	NA931	GE Healthcare (Chicago, IL)
Amicon Ultra spin columns	UFC200324	Merck (Darmstadt, Germany)
LAS-3000 mini		Fujifilm (Tokyo, Japan)

BCA = bicinchoninic acid; CCK-8 = cell counting kit-8; Col2A1 = collagen type II alpha 1; CRISPR–Cas9 = clustered regularly interspaced short palindromic repeats–CRISPR-associated protein 9; DAPI = 4',6-diamidino-2-phenylindole; DMEM = Dulbecco's modified Eagle's medium; FBS = fetal bovine serum; GSDMD = gasdermin-D; IL-1β = interleukin-1 beta; LC3 = light chain 3; MMP = matrix metalloproteinase; mTOR = mammalian target of rapamycin; p16/INK4a = p16/inhibitor of CDK4; p21/WAF1/CIP1 = p21/wild-type p53-activated fragment 1/CDK-interacting protein 1; p62/SQSTM1 = p62/sequestosome 1; p70/S6K = p70/ribosomal S6 kinase; PARP = poly (ADP-ribose) polymerase; PVDF = polyvinylidene difluoride; RAPTOR = regulatory-associated protein of mTOR; RICTOR = rapamycin-insensitive companion of mTOR; RNAi = RNA interference; SA-β-gal = senescence-associated beta-galactosidase; SDS = sodium dodecyl sulfate; siRNA = small interfering RNA; TIMP = tissue inhibitor of metalloproteinases.

Table S2. List of siRNA sequences used.

siRNA			Sequence (5' to 3')
mTOR	No. 1	Sense	AAGCACCUCUCGGAGUUCCA
		Anti-sense	UGGAACUCCGAGAGGUGCUU
	No. 2	Sense	GCUGGGUGCUGACCGCAAUG
		Anti-sense	CAUUGCGGUCAGCACCCAGC
RAPTOR	No. 1	Sense	CUCCUUGGCGUUGCGACGUA
		Anti-sense	UACGUCGCAACGCCAAGGAG
	No. 2	Sense	GUCCUUACGUCGCAACGCCA
		Anti-sense	UGGCGUUGCGACGUAAGGAC
RICTOR	No. 1	Sense	UGACAGCUUCUUUGUGAUAU
		Anti-sense	AUAUCACAAAGAAGCUGUCA
	No. 2	Sense	CCAAAAUGUGGCCAGAUUGC
		Anti-sense	GCAAUCUGGCCACAUUUUGG
Negative control		Sense	CCUAAGGUUAAGUCGCCCUCG
		Anti-sense	CGAGGGCGACUUAACCUUAGG

mTOR = mammalian target of rapamycin; RAPTOR = regulatory-associated protein of mTOR; RICTOR = rapamycin-insensitive companion of mTOR; siRNA = small interfering RNA.

Table S3. List of CRISPR–Cas9 guide RNA sequences used.

CRISPR–Cas9 guide RNA			Sequence (5' to 3')
mTOR	No. 1	Sense	AAGCACCTCTCGGAGTTCCA
	No. 2	Sense	GCTGGGTGCTGACCGCAATG
	No. 3	Sense	GCCAGTCCTCTACGATACGC
RAPTOR	No. 1	Sense	CTCCTTGGCGTTGCGACGTA
	No. 2	Sense	GTCCTTACGTCGCAACGCCA
	No. 3	Sense	TCAGCTGCCGACGATCATCG
RICTOR	No. 1	Sense	TGACAGCTTCTTTGTGATAT
	No. 2	Sense	CCAAAATGTGGCCAGATTGC
	No. 3	Sense	ATGTTCGTCGGAGTAACCAA
Negative control		Sense	GTGTAGTTCGACCATTCGTG

CRISPR–Cas9 = clustered regularly interspaced short palindromic repeats–CRISPR-associated protein 9; mTOR = mammalian target of rapamycin; RAPTOR = regulatory-associated protein of mTOR; RICTOR = rapamycin-insensitive companion of mTOR.
